# A phase separation hypothesis for the biological function of PrP: the role of multivalent interactions at the plasma membrane

**DOI:** 10.1042/BST20253081

**Published:** 2026-01-28

**Authors:** Maria Heloisa Freire, Rafael Linden, Yraima Cordeiro

**Affiliations:** 1 Faculty of Pharmacy, Federal University of Rio de Janeiro, Rio de Janeiro, RJ, 21941-902, Brazil; 2 Carlos Chagas Filho Biophysics Institute, Federal University of Rio de Janeiro, Rio de Janeiro, RJ, 21941-902, Brazil

**Keywords:** biomolecular condensate, multivalency, phase separation, prion protein, protein ligands, Cell membrane

## Abstract

Although the prion protein (PrP) is well established as the etiological agent of transmissible spongiform encephalopathies, its biological function remains under debate. Native, cellular PrP (PrP^C^) is a glycosylphosphatidylinositol-anchored protein that interacts with various proteins and other molecular ligands at the cell surface, triggering diverse cellular responses such as neuritogenesis and neuroprotection. PrP^C^ has been proposed to act as a scaffolding protein, facilitating the assembly of multicomponent complexes at the membrane, with signal transduction occurring through the recruitment of transmembrane proteins. Recent findings demonstrate that PrP undergoes phase separation (PS) *in vitro* and *in cellulo*, mostly driven by its multivalency, intrinsically disordered N-terminal domain, and ability to bind polyanions. Considering recent data showing that membrane multicomponent complexes may assemble through PS, we discuss the possible formation of biomolecular condensates containing PrP at the membrane in light of previously described PrP protein ligands. In this mini-review, we examine PrP’s interactions with key ligands such as epidermal growth factor receptor, apolipoprotein E, amyloid β oligomers, α-synuclein, N-methyl-D-aspartate receptor, postsynaptic density protein 95, integrin β1, and tau, assessing their relevance in PS-mediated condensate formation. These proteins were selected based on their direct or indirect interaction with PrP, biological effects, presence in a membrane environment, and evidence of participation in biomolecular condensates. Based on current evidence, we propose that PS may be a fundamental mechanism underlying PrP’s biological role in the membrane.

## Introduction

The prion protein (PrP) is a conserved protein found in all mammalian species, including humans [1]. Most studies on PrP focus on transmissible spongiform encephalopathies, fatal neurodegenerative diseases caused by an altered form of the cellular PrP (PrP^C^), known as scrapie PrP (PrP^Sc^). The primary difference between PrP^C^ and PrP^Sc^ lies in their secondary structure composition: PrP^Sc^ is enriched in β-sheets and forms aggregated deposits in the central nervous system [2,3]. While significant progress has been made in understanding the conformational conversion of PrP^C^ into PrP^Sc^, many gaps remain regarding both its pathological mechanisms and the physiological function of PrP^C^.

PrP^C^ is predominantly anchored to the cell surface via a glycosylphosphatidylinositol (GPI) anchor, and it can exist in di-, mono-, or non-glycosylated forms [1]. Human PrP is composed of two well-defined domains: an unstructured N-terminal domain (residues 23–120) and a globular C-terminal domain (121–230) consisting of three α-helices and two β-strands [4,5]. PrP^C^ interacts with a wide range of macromolecules, primarily through its N-terminal intrinsically disordered region (IDR). Among these macromolecules, several protein ligands have been identified, with functional interactions linked to neuroprotection, neuritogenesis, cell differentiation, and memory formation [6]. Given its multivalency (i.e. the ability to engage in different interactions with various ligands), its IDR domain, and its localization at the plasma membrane, PrP^C^ is proposed to function as a signaling platform [7,8]. In this role, PrP^C^ would sequentially recruit different ligands, ultimately leading to intracellular signal transduction and the observed biological effects.

The physicochemical properties of PrP suggest that it may drive phase separation (PS) within the cellular environment [5]. PS is a physical process underlying the formation of membrane-less organelles, such as the nucleolus, stress granules, and P-granules [9,10]. The formation of biomolecular condensates in the cytosol and nucleus typically requires scaffold proteins with IDRs capable of driving PS, often in conjunction with RNAs and RNA-binding proteins [11]. Recent studies, including those from our research group, have characterized the liquid–liquid and liquid-to-solid phase transitions of PrP *in vitro*, both in the absence of additional ligands (homotypic PS) and in the presence of ligands (heterotypic PS) [12–15]. These findings suggest that interactions with physiological PrP ligands may trigger PS as part of its functional role, and that, depending on the ligand, this phenomenon could precede a liquid-to-solid transition associated with pathological dysfunction [12,14,16].

Given that the majority of the cellular PrP^C^ pool is anchored to the plasma membrane [17], PS should also be considered within the membrane environment. Although PS was not observed in an *in vitro* model using recombinant PrP linked via GPI to a synthetic membrane (supported lipid bilayers) [18], it is increasingly recognized that multivalent interactions among transmembrane proteins and their ligands can drive PS at the membrane [19–21]. These membrane-associated condensates have been linked to signal transduction processes [20]. Given that PrP forms dynamic clusters at the plasma membrane [14], and participates in multivalent interactions during signaling, it is plausible that membrane-anchored PrP may engage in PS under specific physiological or pathological conditions. This highlights the need for a detailed investigation into phase transitions involving membrane-bound PrP in the presence of its ligands.

**Figure 1:**
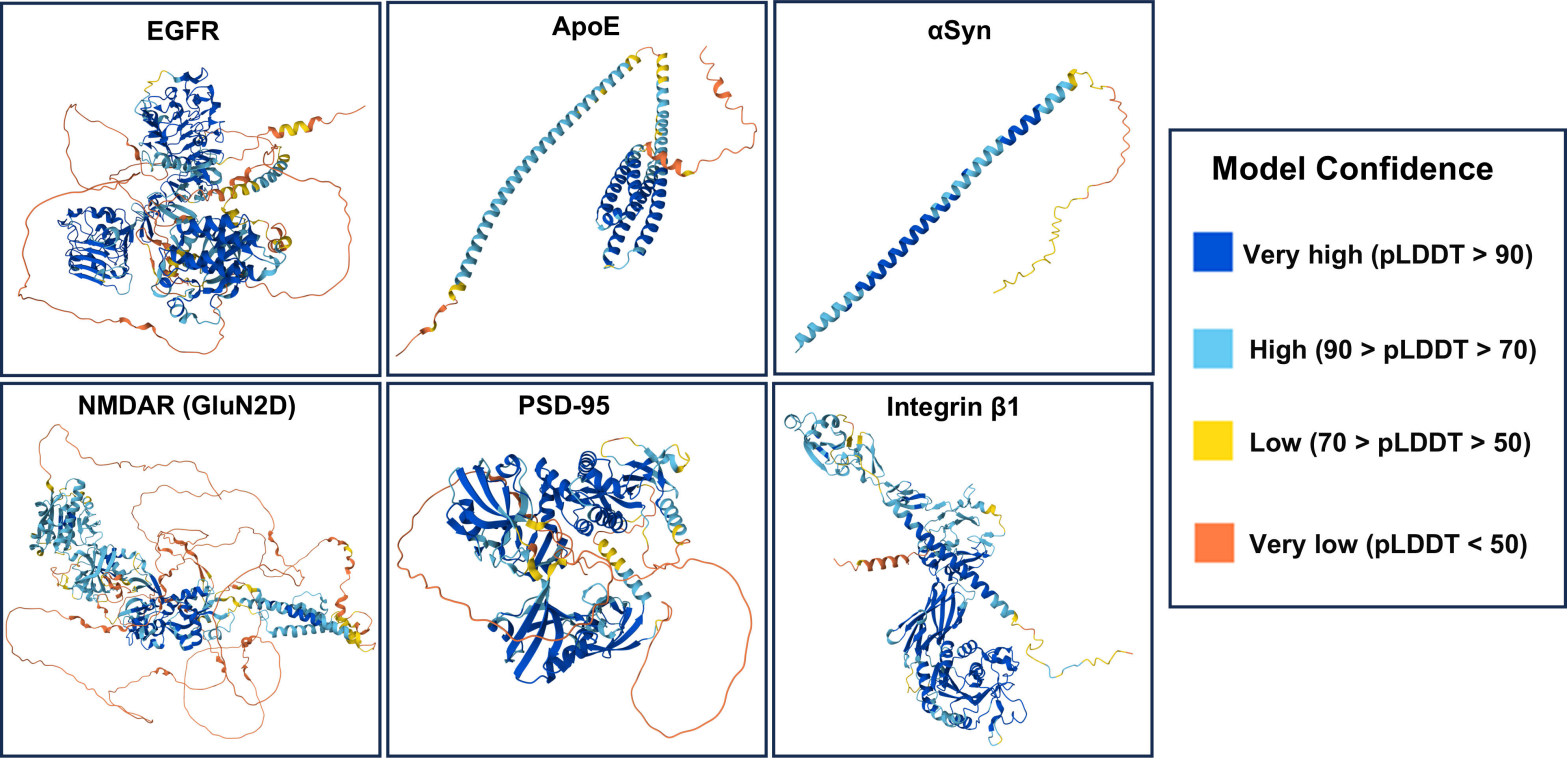
AlphaFold structural models of membrane-associated PrP^C^ ligands involved in phase separation with reliably predicted structures. Full-length models are shown for the following proteins with their corresponding UniProt IDs and residue lengths: epidermal growth factor receptor (EGFR, P00533, 1210 aa), apolipoprotein E (ApoE, P02649, 317 aa), α-synuclein (P37840, 140 aa), the NMDAR subunit GluN2D (O15399, 1336 aa), postsynaptic density protein 95 (PSD-95, P78352, 724 aa), and integrin β1 (P05556, 798 aa). All models are colored by the predicted local distance difference test (pLDDT), where blue indicates high confidence and orange indicates very low confidence. A common feature across all ligands is the coexistence of well-folded, globular domains (blue/cyan) with extensive segments of low-confidence prediction (yellow/orange), reflecting the prevalence of IDRs that are often critical for phase separation.

In this review, we discuss the potential biological roles of PrP^C^ in the context of PS and protein–ligand interactions, based on the following criteria: (i) direct or indirect interaction with PrP, (ii) biological effects, (iii) presence in a membrane environment, and (iv) evidence of participation in biomolecular condensates as either a scaffold or client protein. PrP ligands that meet these criteria include the epidermal growth factor receptor (EGFR), apolipoprotein E (ApoE), amyloid β oligomers (AβOs), α-synuclein (αSyn), N-methyl-D-aspartate receptor (NMDAR), postsynaptic density protein 95 (PSD-95), integrin β1, and tau (Table 1; Figure 1). We will discuss recent findings on these proteins in the context of their interactions with PrP, exploring the hypothesis that PS at the membrane contributes to the PrP biological role.

**Table 1:** Plasma membrane PrP ligands involved in phase separation

Ligand protein	Interaction with PrP (evidence)	Biological context of interaction	PS evidence	References
Epidermal growth factor receptor (EGFR)	Co-IP; colocalization; lipid–raft association; functional assays	ERK/Akt-dependent neuritogenesis and cell proliferation	Heterotypic EGFR–Grb2 PS on Ni–NTA bilayers	[22–26]
Apolipoprotein E (ApoE)	ELISA; Co-IP; pull-down	Absence of ApoE accelerates prion pathology	Homotypic ApoE2 PS under redox conditions	[27–29]
Amyloid β oligomer (AβO)	Cell binding assay; pull-down; SPR; EPR	PrP^C^-dependent synaptic dysfunction: LTP inhibition, altered NMDAR signaling, spine loss	AβOs homotypic PS; AβO-induced heterotypic hydrogel-like condensates of PrP^C^ at the plasma membrane	[15,30–35]
α-synuclein (αSyn)	Co-IP; Western blot; colocalization	PrP-dependent synaptic dysfunction (LTP inhibition; Fyn/GluN2B signaling alteration)	αSyn homotypic PS; PrP–αSyn heterotypic PS with liquid-to-solid transition and amyloid conversion	[36–38]
N-methyl-D-aspartate receptor (NMDAR)	Co-IP; colocalization	Neuroprotection via inhibition of excitotoxicity	Heterotypic NMDAR GluN2B C-terminal domain PS with PSD-95/SynGAP	[39,40]
Postsynaptic density protein 95 (PSD-95)	Co-IP; Western blot	Protection against excitotoxicity	PSD-95 homotypic PS at acidic pH; heterotypic PS with synaptic partners	[41–43]
β1 integrin	Indirect association via caveolin-1; Co-IP with caveolin-1 after PrP activation	Integrin-dependent PrP signaling driving FAK/caveolin-1 activation and neuritogenesis	Recruited as a client protein into FAK–paxillin–p130Cas adhesion condensates	[44–46]
Tau	Pull-down; ELISA; Co-IP	Extracellular tau impairs synaptic function through cell-surface PrP^C^; PrP^C^-dependent uptake of extracellular tau fibrils	Tau homotypic PS *in vitro* and in neurons; heterotypic tau–PrP condensates	[47–52]

LTP, long-term potentiation. SPR, surface plasmon resonance. EPR, electron paramagnetic resonance. Co-IP, co-immunoprecipitation. Ni–NTA, nickel–nitrilotriacetic acid. PS, phase separation.

## Membrane-associated protein ligands of PrP involved in phase separation

### Epidermal growth factor receptor (EGFR)

EGFR is a transmembrane tyrosine kinase receptor that binds multiple extracellular ligands and regulates differentiation, proliferation, survival, and tissue repair in epithelial cells, fibroblasts, and neural progenitors [53].

PrP^C^–EGFR interaction has been confirmed by co-immunoprecipitation (Co-IP), immunofluorescence colocalization, dot blot, and functional assays, occurring predominantly at the plasma membrane [22–25]. PrP^C^ colocalizes with EGFR within lipid rafts [24,25], and this association modulates extracellular signal–regulated kinase/protein kinase B (Akt) (ERK/Akt) signaling pathways, promoting neuritogenesis [25]. It was further demonstrated that PrP^C^ engages the adaptor protein Grb2 (growth factor receptor-bound protein 2) and the active kinase p-Src to activate ERK/Akt and support EGFR-dependent proliferative responses [24].

EGFR undergoes PS with Grb2, as demonstrated using its cytoplasmic tail anchored to a supported lipid bilayer via a His-tag bound to nickel–nitrilotriacetic acid (Ni–NTA) lipids [26].

Similarly to EGFR, low-density lipoprotein receptor-related protein 1 (LRP1) is also localized within lipid rafts. Within these microdomains, LRP1 interacts with PrP^C^ and modulates downstream signaling pathways involving NMDARs, Src family kinases (SFKs), Trk (tropomyosin receptor kinase) receptors, and ERK1/2 [54,55].

**Figure 2:**
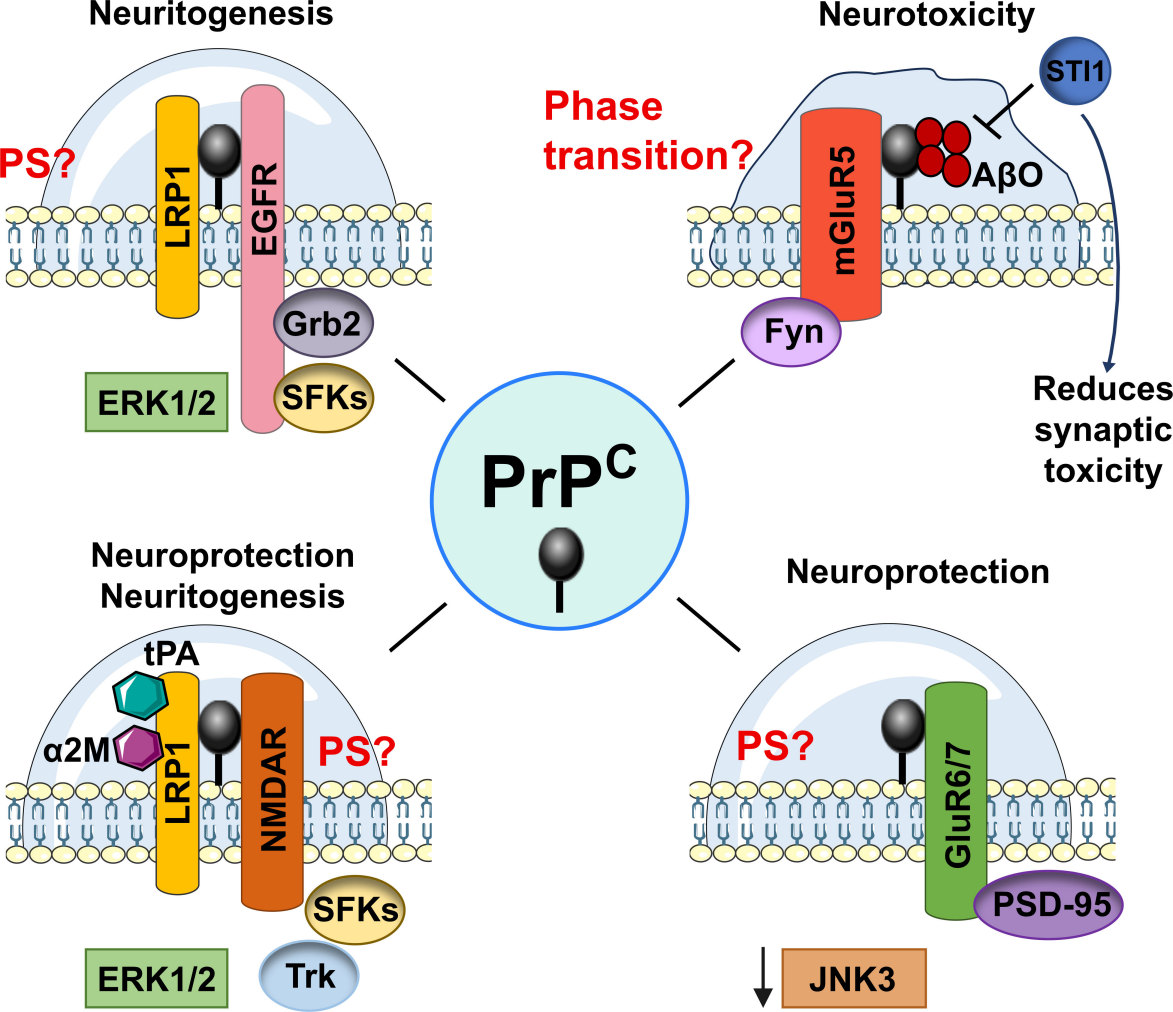
Functional PS hypothesis of PrP^C^ at the plasma membrane through multivalent interactions with protein ligands. PrP^C^ forms multivalent complexes at the plasma membrane with different protein partners, modulating signaling pathways associated with neuroprotective or neurotoxic outcomes. **Top left**: PrP^C^ interacts with LRP1 and EGFR; the latter recruits the adaptor protein Grb2 and activates SFKs, leading to the activation of the ERK1/2 signaling pathway. The multivalency of these interactions may promote condensate formation through PS at the membrane. **Bottom left**: PrP^C^ forms complexes with NMDAR and LRP1, with LRP1 also associating with α2M and tPA. This assembly is linked to SFK activation and downstream Trk and ERK1/2 signaling pathways, promoting neuroprotection and neuritogenesis, potentially involving PS. **Top right**: The interaction of PrP^C^ with mGluR5 and AβO activates the Fyn kinase in a context associated with neurotoxicity. The co-chaperone STI1 can compete with AβOs, negatively modulating this pathway. The formation of this complex suggests the possibility of a phase transition with pathological consequences. **Bottom right**: PrP^C^ interacts with GluR6/7 and PSD-95 at the postsynaptic density, preventing the overactivation of the JNK3 (c-Jun N-terminal kinase 3) pathway. In the absence of PrP^C^, enhanced interaction between PSD-95 and GluR6/7 promotes JNK3-mediated neurotoxicity. Sustained JNK3 activation may disrupt protein homeostasis at the synapse, potentially leading to PS dysregulation and the formation of solid-like protein aggregates. PS, phase separation; tPA, tissue plasminogen activator; α2M, α2-macroglobulin; STI1, stress-inducible phosphoprotein 1; JNK3, c-Jun N-terminal kinase 3.

The presence of PrP, EGFR, and LRP1 within lipid rafts increases the local density of signaling receptors and adaptors, including Grb2 and SFKs, which may facilitate the assembly of a higher order signaling platform. The multivalency of interactions concentrated in these microdomains could further promote the formation of membrane-associated condensates, a mechanism that may enhance ERK1/2 signaling and thereby support neuritogenesis (Figure 2, top left).

### Apolipoprotein E (ApoE)

ApoE is a lipoprotein involved in cholesterol and lipid transport, playing an essential role in neuronal homeostasis and lipid metabolism [56].

Full-length recombinant human PrP interacts *in vitro* with all three ApoE isoforms (E2, E3, and E4), binding to the ApoE N-terminal region (residues 1–194) via its own N-terminal segment (residues 23–90) [27]. This interaction likely occurs at the plasma membrane, possibly within caveolae-like domains, where PrP^C^ conversion to PrP^Sc^ may take place [27]. ApoE plays a critical role in prion disease progression; its absence accelerates pathology, increases neuronal loss, and heightens neuroinflammation [28]. Additionally, ApoE modulates the course of other neurodegenerative disorders by influencing protein aggregation, clearance, and immune responses [57].

ApoE2 has been shown to undergo PS regulated by redox conditions, due to cysteine oxidation at positions 112 and 158, leading to condensate formation in the retinal pigment epithelium. In contrast, ApoE4, which contains arginine residues at these positions and thus lacks oxidizable thiol groups, is resistant to this process [29].

These findings suggest that ApoE and PrP may co-partition into phase-separated membrane domains, potentially influencing PS at the plasma membrane. Given that ApoE2 undergoes PS in a redox-dependent manner [29] and that PrP has been implicated in PS [12,13,58], their interaction could contribute to the formation of dynamic membrane-associated condensates. Over time, age-related lipid alterations, oxidative stress, and ApoE modifications may destabilize these phase-separated domains, leading to condensate aging, increased viscosity, and eventual aggregation. This transition from a dynamic to a pathological state could facilitate the conversion of PrP^C^ to PrP^Sc^, thereby contributing to neurodegeneration.

### Amyloid β oligomer (AβO)

AβOs are soluble aggregates of the Aβ peptide, which is derived from the amyloid precursor protein through proteolytic cleavage by β- and γ-secretases [59].

AβOs bind to two distinct regions of PrP^C^: the 95–105 segment and the N-terminal residues 23–27, as demonstrated by cell-binding assays, pull-down experiments, surface plasmon resonance (SPR), and electron paramagnetic resonance (EPR) [30,31]. PrP^C^ functions as a receptor for AβOs, mediating synaptotoxic effects such as reduced synaptic plasticity and inhibition of long-term potentiation (LTP) [30]. Several key modulators influence this interaction. PrP^C^ forms a membrane complex with metabotropic glutamate receptor 5 (mGluR5) that facilitates AβOs binding and toxic signaling [32]. Stress-inducible phosphoprotein 1 (STI1) binds PrP^C^ at residues 113–128, sterically blocking the adjacent AβO site and reducing AβO-induced toxicity [33]. Additionally, AβOs binding to PrP^C^ activates Fyn kinase, triggering phosphorylation of the N-methyl-D-aspartate receptor subunit 2B (GluN2B), dendritic spine loss, and impaired synaptic function [34]. Together, these findings highlight PrP^C^ as a central mediator of AβO-induced neurotoxicity, co-ordinating membrane interactions with mGluR5 and STI1 and activating downstream Fyn signaling.

AβOs undergo PS *in vitro*, driven by hydrophobic interactions and modulated by salt conditions. The same study demonstrates that PS of AβOs redirects their aggregation pathway toward amyloid fibril formation [35]. PrP^C^ also undergoes PS and forms a hydrogel upon interaction with AβOs; this hydrogel dissolves in the presence of excess AβOs and promotes the recruitment of mGluR5 to the membrane [15].

Building on the established role of PrP^C^–mGluR5 complexes in AβO-related synaptic dysfunction [32], AβO binding to PrP^C^ may stabilize a condensed state in which AβOs become immobilized while PrP^C^ retains relative mobility, potentially disrupting membrane dynamics and promoting the formation of a hydrogel-like assembly. Within this context, the PrP^C^–AβO complex embedded in the hydrogel structure recruits and immobilizes mGluR5 at the plasma membrane, further enhancing synaptic toxicity [15]. Although still speculative, this hydrogel may represent a critical intermediate in the transition from physiological to pathological states.

In this context, STI1 has been identified as a neuroprotective modulator, competing with AβOs for PrP^C^ binding and attenuating its toxicity [33]. If the stabilization of the hydrogel plays a central role in promoting AβO aggregation, STI1 may help prevent this pathological progression. However, an imbalance in the stoichiometry among PrP^C^, AβOs, and STI1 could instead favor hydrogel persistence, potentially contributing to neurodegenerative mechanisms (Figure 2, top right).

### α*-*synuclein (αSyn)

αSyn is primarily an intrinsically disordered protein in the cytosol, but it adopts transient α-helical structures upon membrane binding. It plays a key role in regulating synaptic vesicle trafficking and calcium homeostasis at presynaptic terminals. However, its misfolding into β-sheet-rich aggregates is associated with the pathogenesis of Parkinson’s disease [60].

PrP^C^ specifically binds to the oligomeric form of αSyn through its 93–109 region, forming a signaling complex with Fyn and GluN2B. Inhibition of this region using the 6D11 antibody effectively blocked the toxic effects of αSyn on synaptic plasticity [36]. Additionally, HEK293 cells transfected with PrP^C^ exhibited increased αSyn binding to the membrane, whereas deletion of the PrP^C^ ‘charged cluster’ domain (95–110) significantly reduced this interaction [61].

αSyn undergoes PS both *in vitro* and *in cellulo*, forming dynamic condensates that evolve into solid aggregates [37]. PrP and monomeric αSyn also coacervate directly through domain-specific electrostatic interactions, generating highly dynamic heterotypic condensates that undergo liquid-to-solid transitions and promote amyloid conversion [38]. In neuronal cells, oligomeric αSyn engages PrP^C^ to assemble a multivalent complex involving Fyn, GluN2B, and mGluR5, a configuration associated with impaired synaptic plasticity [36]. Based on these findings, it is plausible that PrP–αSyn interaction at the plasma membrane gives rise to dynamic condensates that, depending on the conformational state of αSyn and the surrounding molecular environment, may evolve into more rigid assemblies and solid aggregates. These findings suggest that PS-mediated PrP–αSyn condensates at the membrane could act as precursors to pathological aggregation and thereby contribute to neurodegenerative processes.

### N-methyl-D-aspartate receptor (NMDAR)

NMDAR is an ionotropic glutamate receptor essential for excitatory neurotransmission, synaptic plasticity, and processes such as learning and memory [62].

A Co-IP assay revealed that PrP^C^ associates with the N-methyl-D-aspartate receptor subunit 2D (GluN2D), forming a signaling complex in hippocampal homogenates. Additionally, the colocalization of PrP^C^ and GluN2D at the cell surface of hippocampal neurons was confirmed by immunofluorescence [39].

The C-terminal domain of the GluN2B subunit of the NMDAR undergoes PS *in vitro* when combined with PSD-95 and Synaptic Ras GTPase-activating protein (SynGAP). This was demonstrated in assays performed at neutral pH and physiological ionic strength, using purified recombinant proteins incubated at 25°C [40].

The interaction of PrP with NMDAR and its association with PSD-95 suggest a potential role in the organization of dynamic synaptic complexes. PrP also binds to LRP1 [54,55], a multifunctional receptor that interacts with signaling activators such as tissue plasminogen activator (tPA) and α2-macroglobulin (α2M) [55]. Soluble PrP derivatives engage the lipid raft-localized LRP1/NMDAR complex, activating ERK1/2 phosphorylation through SFKs and Trk receptors, thereby promoting neuritogenesis and Schwann cell migration [55]. LRP1 also forms a complex with NMDAR and PSD-95 in neurons and co-immunoprecipitates with PSD-95, supporting its role in postsynaptic protein networks [63]. Given that PrP undergoes PS and that the C-terminal domain of the GluN2B subunit of the NMDAR forms condensates with PSD-95, together with the evidence that LRP1 also associates with PSD-95 and NMDAR, this network of multivalent interactions could substantially increase local interaction density at the plasma membrane. Such multivalency may facilitate condensate-like organization of signaling complexes, thereby amplifying SFK–ERK1/2–Trk–dependent pathways that support neuritogenesis, synaptic stability, and neuroprotection (Figure 2, bottom left).

### Postsynaptic density protein 95 (PSD-95)

PSD-95 is a scaffold protein essential for organizing excitatory synapses, localized within the postsynaptic density . Anchored to the membrane via palmitoylation, PSD-95 stabilizes and links neurotransmitter receptors, such as NMDAR and glutamate receptor 6/7 (GluR6/7), to intracellular signaling proteins and the cytoskeleton [41,64].

PrP^C^ interacts with PSD-95 at the postsynaptic membrane, modulating neurotransmission through GluR6/7. This interaction was confirmed by Co-IP of hippocampal extracts enriched for the postsynaptic density fraction. Western blot analysis detected PSD-95, PrP^C^, and GluR6/7, suggesting that PrP^C^ contributes to the organization of this complex at the membrane. Although PrP^C^ is anchored to the extracellular leaflet and PSD-95 is intracellular, their association is likely mediated by transmembrane proteins such as GluR6/7. Functionally, PrP^C^ appears to regulate the association of GluR6/7 with PSD-95, limiting this interaction and thereby suppressing downstream c-Jun N-terminal kinase 3 (JNK3) activation, which protects against excitotoxicity-induced neuronal death [41].

PSD-95 undergoes homotypic PS under acidic conditions (pH 5.4), forming condensates through self-interaction [42]. It also phase-separates heterotypically in the presence of synaptic membrane-associated proteins involved in excitatory neurotransmission [43]. The efficiency of PS is strongly influenced by the multivalency of its interacting partners, with higher order oligomeric forms promoting condensation more effectively than monomeric ones [42].

The interaction of PrP^C^ with PSD-95 and GluR6/7 at the PSD suggests a potential role in the modulation of PS at the plasma membrane. Since PSD-95 can undergo both homotypic and heterotypic PS, its association with PrP^C^ may promote the formation of dynamic biomolecular condensates at the extracellular–intracellular interface. These condensates could contribute to organizing the spatial distribution of receptors such as GluR6/7 and NMDAR, possibly restricting excessive PSD-95–GluR6/7 interactions and thereby attenuating JNK3 pathway activation. In the absence of PrP^C^, dysregulation of this interaction may lead to sustained JNK3 activation, potentially disrupting condensate dynamics and promoting pathological phase transitions (Figure 2, bottom right).

### Integrin β1

Integrins are transmembrane proteins that function as cell adhesion receptors, connecting the intracellular cytoskeleton to the extracellular microenvironment. They are composed of two subunits, α and β, which combine to generate different functional heterodimers [65].

In PC12 cells endogenously expressing PrP, cross-linking of the N-terminal domain with the SAF32 antibody functionally activated PrP, leading to phosphorylation of caveolin-1 and focal adhesion kinase (FAK). This activation induced β1 integrin reorganization and the formation of adhesion complexes, suggesting that PrP may influence integrin activation and recruitment to specific membrane domains [44].

β1 integrin has been identified as a client protein within condensates formed by PS [45,46]. Although β1 integrin does not undergo PS directly, it is recruited into condensates organized by key adhesion regulators such as FAK, paxillin, and p130Cas (Crk-associated substrate), which act as scaffolds for cell adhesion complexes [66,67].

Thus, it is possible to infer a role for PrP in modulating the PS of focal adhesions, acting as an upstream regulator of FAK activation. Since PrP directly regulates the activation of FAK and caveolin-1 [44], and given that FAK is a critical component in the formation of adhesion condensates [46], PrP may directly modulate the recruitment of β1 integrin into PS compartments, influencing the formation, stability, and organization of cell adhesions.

### Tau

Although primarily a cytoplasmic protein, tau is also released into the extracellular space by neurons in an activity-dependent manner [68]. Extracellular tau exerts both physiological and pathological effects [69], and its aggregation and spread are influenced by lipid membranes and their associated proteins [70].

Tau interacts directly with the N-terminal region of PrP, as demonstrated using recombinant proteins in pull-down, ELISA, and Co-IP assays [47,48]. Subsequent functional studies showed that cell-surface PrP^C^ facilitates the uptake of tau amyloid fibrils, an effect blocked by anti-PrP antibodies [49]. Complementarily, administration of anti-PrP antibodies *in vivo* prevented the impairment of hippocampal synaptic plasticity induced by soluble tau [50].

Tau undergoes PS both *in vitro* and in neurons, rapidly transitioning to gel-like assemblies that lose mobility and initiate pathological aggregation [51]. Tau also forms heterotypic PS with PrP through electrostatic interactions, generating condensates that progressively harden into solid co-aggregates [52].

Together, these observations support the hypothesis that tau–PrP interactions promote the formation of heterotypic condensates in neuronal membrane microdomains, which may directly impair synaptic signaling and/or progressively convert into aggregation-prone structures, thereby linking extracellular tau to early synaptic dysfunction in tauopathies.

## Conclusions

It is proposed that PrP functions as a dynamic platform at the cell surface, capable of organizing multicomponent protein complexes in a regulatable and allosterically modulated manner, leading to distinct functional outcomes [7]. In this context, PS at the plasma membrane could facilitate the formation of PrP clusters, acting as spatial organization hubs for the assembly of proteins and other cellular components.

Several membrane ligands that interact with PrP—including EGFR, ApoE, AβO, αSyn, NMDAR, PSD-95, integrin β1, and tau—are known to undergo or participate in PS. This suggests that PrP may contribute to the formation of membranous biomolecular condensates. While direct evidence of PrP undergoing PS at the cell surface is still lacking, and no PS has been observed in synthetic lipid bilayer systems [18], its interaction with these PS-prone partners points to a potential role as a scaffold or nucleation point for dynamic, multicomponent hubs. Such phase-separated platforms could provide plasticity and responsiveness to PrP-associated complexes, enabling the formation of transient, regulated functional domains at the plasma membrane. These domains may then co-ordinate diverse cellular processes, from neuroprotection and neurogenesis to synaptic dysregulation and excitotoxicity modulation.

**Perspectives**

- Phase separation (PS) has emerged as a potential mechanism for organizing membrane-associated signaling, offering a dynamic mode of concentrating receptors and regulatory factors. Although experimental evidence is still limited, this framework has important implications for understanding both physiological regulation and processes linked to neurodegenerative diseases.
- Although prion protein (PrP) undergoes PS *in vitro*, its occurrence at the plasma membrane remains unclear. It is crucial to distinguish true condensates from glycosylphosphatidylinositol-anchored nanoclusters or lipid raft microdomains, which may lack key PS features like fusion and rapid molecular exchange.
- Determining whether PrP acts as a cofactor or modulator of PS in membrane environments is essential for understanding its role in membrane dynamics and cellular signaling. Investigations using synthetic membranes and cultured cell systems are critical to elucidate whether PrP undergoes PS and to assess its physiological and pathological implications.

## Abbreviations

ApoE: apolipoprotein E
AβO: amyloid β oligomer
Co-IP: co-immunoprecipitation
EGFR: epidermal growth factor receptor
EPR: electron paramagnetic resonance
FAK: focal adhesion kinase
GPI: glycosylphosphatidylinositol
GluR6/7: glutamate receptor 6/7
Grb2: growth factor receptor-bound protein 2
IDR: intrinsically disordered region
JNK3: c-Jun N-terminal kinase 3
LRP1: low-density lipoprotein receptor–related protein 1
LTP: long-term potentiation
NMDAR: N-methyl-D-aspartate receptor
Ni–NTA: nickel–nitrilotriacetic acid
PS: phase separation
PSD-95: postsynaptic density protein 95
PrP: prion protein
PrP^C^: cellular prion protein
PrP^Sc^: scrapie PrP
SFKs: Src family kinases
SPR: surface plasmon resonance
STI1: stress-inducible phosphoprotein 1
SynGAP: Synaptic Ras GTPase-activating protein
Trk: tropomyosin receptor kinase
mGluR5: metabotropic glutamate receptor 5
p130Cas: Crk-associated substrate
pLDDT: predicted local distance difference test
tPA: tissue plasminogen activator
α2M: α2-macroglobulin
αSyn: α-synuclein
